# A Framework for Selecting Zebrafish Models of Metabolic Diseases: Modeling Strategies, Phenotypic Validation, Mechanistic Investigation, and Efficacy Evaluation

**DOI:** 10.3390/biomedicines14081857

**Published:** 2026-08-18

**Authors:** Yiqi Zhu, Ji Ma, Chao Song

**Affiliations:** Faculty of Life Science and Technology & The Affiliated Anning First People’s Hospital, Kunming University of Science and Technology, Kunming 650302, China

**Keywords:** zebrafish, metabolic disease, disease model, model selection

## Abstract

Metabolic diseases have diverse etiologies, prolonged courses, and multiorgan involvement, making selection of animal models matched to the research objective and disease stage essential. Zebrafish develop rapidly, are genetically tractable, and support in vivo imaging and high-throughput screening, enabling studies of obesity and dyslipidemia, diabetes and its complications, fatty liver disease, atherosclerosis, and inherited metabolic diseases. However, zebrafish models differ in developmental stage, induction conditions, phenotypic evidence, and fidelity to human disease, and a single abnormal phenotype rarely supports a complete disease designation or mechanistic conclusion. This review compares dietary, chemical, genetic, and combined models in terms of modeling characteristics, representative phenotypes, applications, and limitations. It summarizes morphological, biochemical, and functional assays, in vivo imaging, omics, and automated quantification for phenotypic validation; discusses metabolic imbalance, mitochondrial dysfunction and oxidative stress, inflammation and immunometabolism, and interorgan crosstalk; and evaluates drug-screening applications and translational limitations. We propose a model-selection framework based on research objective and disease stage, developmental stage, modeling strategy, and core-phenotype validation. Minimum validation criteria define appropriate disease terminology and inference. A larval rapid-screening-to-adult-systemic-validation strategy, multilevel outcome assessment, and cross-model validation may improve the reliability and translational value of zebrafish research on metabolic diseases.

## 1. Introduction

Metabolic diseases include obesity, diabetes, fatty liver disease, atherosclerosis, and inherited metabolic disorders. Their development and progression commonly involve dysregulated glucose and lipid metabolism, mitochondrial dysfunction, oxidative stress, chronic inflammation, and interorgan metabolic crosstalk [1,2,3,4]. These diseases often have complex etiologies, prolonged courses, and multiorgan involvement, and the same disease may present distinct metabolic, histological, and functional abnormalities at different stages [5,6]. Animal models should therefore not only generate measurable disease-related phenotypes but also match the specific research question in terms of inducing factors, disease stage, and strength of evidence.

Zebrafish combine the organ systems and fundamental metabolic regulation of a vertebrate with a small body size, high fecundity, rapid development, and convenient genetic manipulation [7]. The transparency of embryos and larvae can be combined with organ- or cell-specific fluorescent reporter lines to visualize dynamic changes in the liver, pancreas, vasculature, and immune cells in vivo; standardized lipid-droplet imaging also enables quantitative assessment of hepatic lipid deposition in larvae [8,9]. By contrast, adult zebrafish have more mature organs and metabolic systems and are suitable for long-term dietary or genetic interventions followed by systematic assessment using histopathology, biochemical measures, and organ-function outcomes [6,10]. Larvae are therefore well suited to early phenotypic observation and rapid screening, whereas adults are better suited to chronic disease modeling and systemic validation; the two stages are strongly complementary.

Dietary induction, chemical induction, genetic manipulation, and combined interventions have been used to establish a wide range of zebrafish models of metabolic disease. However, studies vary considerably in developmental stage, induction dose, treatment duration, feed composition, and phenotypic endpoints. More importantly, individual models generally reproduce only selected components of a disease process. For clarity, throughout this review, “disease model” is used as a general term for an experimental system intended to reproduce relevant aspects of a human metabolic disease, whereas the qualifier “-like” is used more cautiously when referring to phenotypes or pathological changes that reproduce selected or early features of the corresponding disease. Thus, the use of “-like” indicates a more limited scope of phenotypic resemblance rather than a categorical distinction in overall model quality. For example, short-term high-glucose exposure primarily reflects a hyperglycemic environment and early glucose-induced injury and cannot be equated directly with chronic diabetes; hepatic lipid deposition alone demonstrates steatosis but cannot independently establish steatohepatitis; and vascular lipid deposition and immune-cell recruitment in larvae mainly represent early atherosclerosis-like changes rather than mature plaques or plaque rupture [2,11,12]. Likewise, inherited metabolic disease models should not be considered successful solely on the basis of morphological or behavioral abnormalities; a complete evidence chain should also include the relevant metabolites, enzyme activity, organ function, and phenotypic rescue.

The central question in zebrafish metabolic-disease research is therefore no longer simply whether an abnormal phenotype can be produced, but what scientific question a model can answer, which disease stage it represents, and how far the available evidence permits conclusions to extend. Model selection should consider etiological relevance, developmental stage, modeling efficiency, fidelity to the disease, core validation endpoints, and the intended downstream translation. Failure to make these distinctions can lead to overly broad disease labels, overinterpretation of mechanisms, or a mismatch between efficacy assessment and model stage.

Accordingly, this review examines obesity and dyslipidemia, diabetes and its complications, nonalcoholic fatty liver disease/nonalcoholic steatohepatitis (NAFLD/NASH), atherosclerosis, and inherited metabolic diseases. For each condition, we compare modeling methods, developmental stages, core phenotypes, appropriate applications, and key limitations, and summarize relevant methods for phenotypic validation, mechanistic research, and therapeutic investigation. Rather than merely cataloguing models, we focus on the research purposes for which each model is suitable, the disease stage it represents, the core evidence required for validation, and the range of disease terminology and conclusions supported by that evidence. On this basis, we propose a framework centered on research objective and disease stage, developmental stage, modeling strategy, and validation of core phenotypes. By linking minimum validation criteria and limits of inference to model selection, disease designation, mechanistic interpretation, and efficacy evaluation, the framework provides a practical basis for the rational selection, standardized validation, and translational use of zebrafish models of metabolic disease.

## 2. Literature Search Strategy

To support the development of this framework, a structured literature search was conducted using PubMed and Web of Science. Studies published from database inception through July 2026 were considered, with particular emphasis on recent original research articles describing zebrafish models of metabolic diseases. Search terms included combinations of “zebrafish” or “*Danio rerio*” with disease-related terms such as “obesity,” “diabetes,” “insulin resistance,” “non-alcoholic fatty liver disease,” “metabolic dysfunction-associated steatotic liver disease,” “steatohepatitis,” and “metabolic syndrome,” together with terms related to model induction, genetic manipulation, phenotype, and validation.

Original studies describing the establishment, characterization, or application of zebrafish metabolic disease models were included when they provided relevant information on model induction methods, major metabolic phenotypes, molecular or histological validation, experimental applications, or model limitations. Reviews and other secondary sources were used primarily to identify additional relevant studies and provide background information. Studies with insufficient methodological or phenotypic information, or those not directly relevant to metabolic disease modeling in zebrafish, were excluded. Reference lists of relevant articles were also screened to identify additional studies.

The literature was synthesized qualitatively to compare model characteristics, validation strategies, applications, and limitations, which collectively informed the model-selection framework proposed in this review.

## 3. Advantages of Zebrafish for Metabolic-Disease Research

### 3.1. Advantages of Larval Models

The principal advantages of larval zebrafish arise from their transparency, external development, small size, and rapid growth. In addition, the use of embryonic and early larval (eleutheroembryonic) zebrafish stages is consistent with the principles of Replacement, Reduction, and Refinement (the 3Rs) and provides an alternative approach that may reduce the use of conventional animal experiments [13]. Embryos and larvae develop externally and remain highly transparent, allowing organ- or cell-specific fluorescent transgenic lines to be used for in vivo visualization of structures and dynamic changes in the liver, pancreas, vasculature, and immune cells [8]. For metabolic phenotypes, lipid staining and live imaging can also be used to quantify and track the distribution of hepatic lipid droplets in larvae [9]. Because larvae are small and develop quickly, they can be cultured and treated in 96- or 384-well plates, require small amounts of test compounds, and permit short experimental cycles, making them suitable for high-throughput screening of candidate drugs, natural products, and bioactive dietary components [14,15]. Adipose tissue and metabolic regulatory systems are still developing at the larval stage, which makes larvae useful for studying early metabolic perturbations and short-term interventions; questions involving mature adipose tissue or chronic disease progression require complementary validation in adults [5,16].

### 3.2. Advantages of Adult Models

Compared with larvae, adult zebrafish are better suited to investigations of adult-onset disease etiology, progression, and long-term intervention [5]. In nutrient-excess models, several weeks of dietary induction can produce systemic metabolic phenotypes such as weight gain, lipid deposition, and dyslipidemia [17]. In adult zebrafish models of NAFLD, sustained dietary intervention can be evaluated using serum lipids, liver-function measures, and histopathology to assess steatosis and liver injury [10]. Adult models are therefore appropriate for integrated assessment of systemic metabolism, histopathological changes, and long-term treatment effects. Their experimental cycles and husbandry costs are higher, however, and conventional pigmented adults have lower external transparency, making in vivo imaging and high-throughput screening less convenient than in embryos or larvae.

### 3.3. Complementarity of Larval and Adult Models

**Figure 1:**
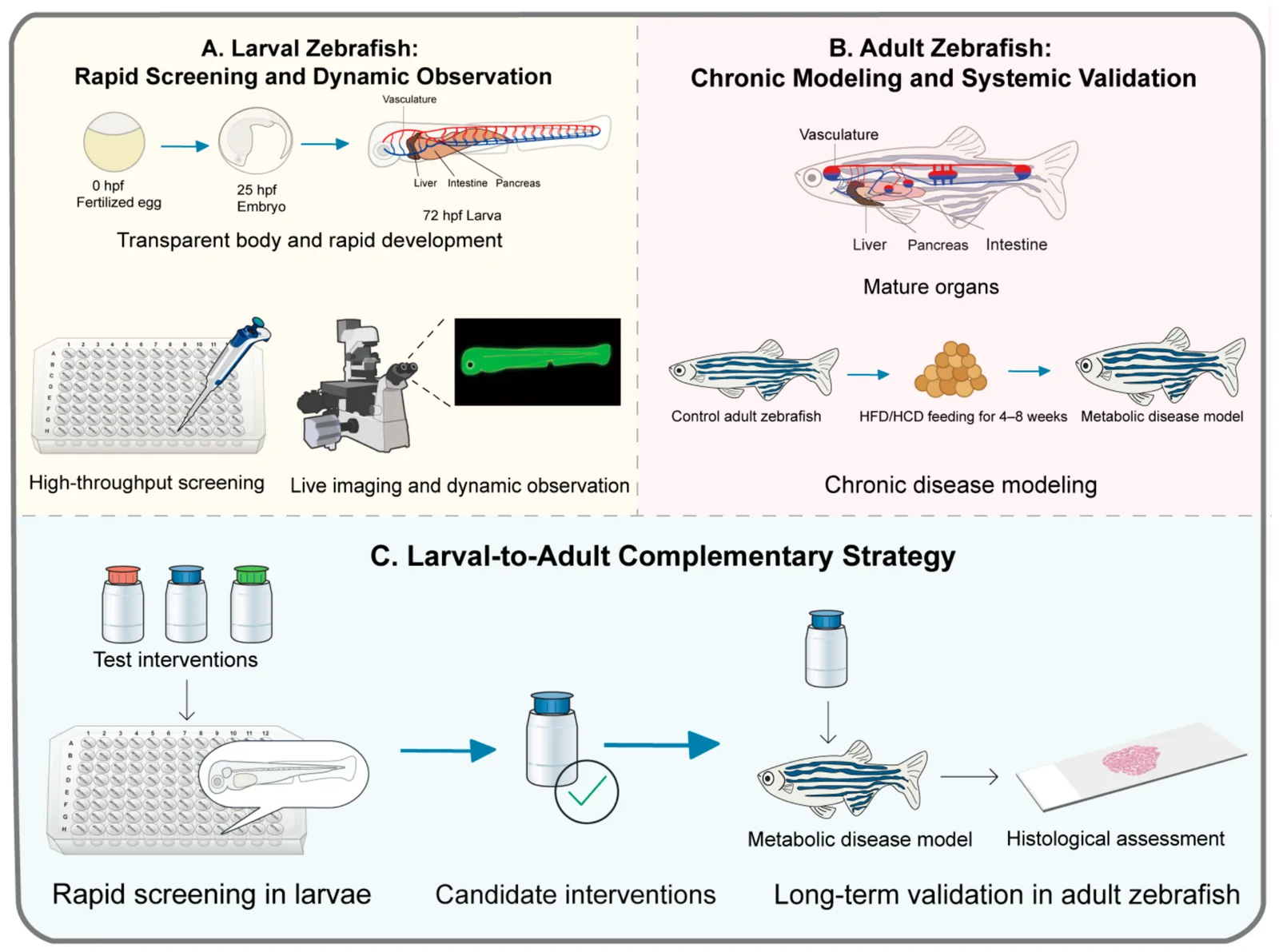
Complementary applications of larval and adult zebrafish in metabolic disease research. (**A**) Larval zebrafish for rapid screening and dynamic observation; (**B**) adult zebrafish for chronic modeling and systemic validation; (**C**) a larval-to-adult strategy integrating rapid screening with long-term validation. The microscope illustration was adapted from NIAID Visual & Medical Arts, NIH BioArt Source (BIOART-000503; creator: Ryan Kissinger), which is in the public domain. The zebrafish vascular system was adapted from Hoareau et al. [18] under the CC BY 4.0 license.

Larval and adult models are complementary primarily because they link successive stages of investigation (Figure 1). Embryos and larvae can be used with automated imaging and multiwell platforms for early phenotypic discovery and screening of candidate interventions [14,15]. The persistence of identified phenotypes can then be evaluated in adult disease models, together with systemic metabolic, histopathological, and organ-function outcomes [5,10]. This larval rapid-screening-to-adult-systemic-validation strategy combines experimental efficiency with the more complete assessment required for chronic metabolic disease.

## 4. Modeling Strategies and Model Selection for Major Metabolic Diseases

Zebrafish models of metabolic disease are established mainly by dietary induction, chemical induction, genetic manipulation, or combined interventions. These approaches serve different research purposes. Diet-induced models are generally more suitable for acquired metabolic diseases such as obesity, dyslipidemia, NAFLD/NASH, and type 2 diabetes. Chemically induced models are often used to rapidly generate phenotypes associated with hyperglycemic injury, pancreatic β-cell damage, or liver injury. Genetic models are better suited to inherited metabolic diseases or pathway abnormalities caused by specific gene defects. Combined interventions can reproduce complex pathological processes involving multiple factors. This section therefore summarizes commonly used methods, representative phenotypes, and appropriate applications by major disease category.

### 4.1. Models of Obesity and Dyslipidemia

Obesity and dyslipidemia are major pathological foundations of metabolic disease and are characterized by excessive fat accumulation, disrupted lipid metabolism, abnormal circulating lipid levels, and ectopic lipid deposition in tissues such as the liver, vasculature, and skeletal muscle [19,20]. Zebrafish models of obesity and dyslipidemia can be established through dietary induction, genetic manipulation, or combined interventions.

Dietary induction is the most widely used strategy and includes overfeeding, high-fat diets (HFDs), and high-cholesterol diets (HCDs). For example, Li et al. established a diet-induced obesity model by overfeeding zebrafish with Artemia: the control group received 5 mg Artemia per day, whereas the overfed group received 60 mg per day. After 9 weeks, overfed zebrafish showed increased body weight, whole-body lipid content, plasma triglycerides, and lipid-droplet deposition in the liver and muscle, supporting the use of this model for obesity and dyslipidemia [17]. Unlike simple overfeeding, HFD models are generally established by increasing the fat content of the feed. Smolińska et al., for example, developed HFD40, a diet containing 40% beef fat. Adult zebrafish fed HFD40 for 8 weeks beginning at 3 months of age developed increased body mass index (BMI), visceral fat accumulation, and hepatic steatosis [21]. HCD models focus more specifically on hypercholesterolemia and disturbed lipoprotein metabolism; an optimized HCD increases apolipoprotein B-containing lipoproteins (ApoB-LPs), plasma cholesterol, and cholesteryl esters and induces fasting-associated hepatic steatosis in larvae [22]. Importantly, the metabolic phenotypes induced by dietary interventions have been reproduced across independent groups using different feeding protocols. Recent studies employing Artemia overfeeding, lipid-enriched diets, or HFD feeding similarly reported increased adiposity, hepatic steatosis, dyslipidemia, and/or metabolic inflammation, supporting the reproducibility of diet-induced zebrafish models of obesity and dyslipidemia [23,24].

Genetic models regulate genes involved in obesity and lipid metabolism through approaches such as transgenic overexpression and gene editing. Tsai et al. generated doxycycline-inducible transgenic zebrafish overexpressing MAX dimerization protein 3 (*mxd3*), a protein-coding gene encoding a MAX-interacting DNA-binding transcriptional regulator, and found that *mxd3* overexpression increased body weight, visceral adiposity, and lipid deposition [25]. Using CRISPR/Cas9, Fei et al. created a knockout model of liver-expressed antimicrobial peptide 2 (*leap2*), a protein-coding gene whose secreted peptide product functions in antimicrobial defense and modulates ghrelin/growth-hormone signaling, in which *leap2* deficiency increased food intake, body weight, and hepatic lipid-droplet accumulation [26]. Zeng et al. established a mutant zebrafish model targeting cordon-bleu WH2 repeat protein-like 1a (*cobll1a*), a protein-coding gene involved in retinoic-acid signaling and lipid homeostasis, and showed that *cobll1a* loss disrupted retinoic-acid signaling, lipid synthesis and catabolism, and lipid transport, leading to lipid accumulation [27]. Despite targeting distinct molecular regulators, these independent genetic studies converge on altered lipid accumulation, body-weight regulation, and/or hepatic lipid metabolism, supporting the utility of genetic manipulation for modeling obesity- and dyslipidemia-related phenotypes in zebrafish [25,26,27].

Combined interventions can also be used to investigate dyslipidemia. For example, an “unhealthy lifestyle” model combining an HFD with sleep deprivation exacerbated lipid deposition and inflammation and was used to study the joint contribution of sleep, diet, and inflammatory signaling to metabolic dysfunction [28]. Such models more closely reflect the multifactorial exposures underlying human obesity and dyslipidemia. However, as this combined HFD–sleep deprivation paradigm has only recently been established, independent replication across laboratories remains limited.

Thus, overfeeding and HFDs primarily model obesity and ectopic lipid deposition caused by excess energy intake; HCDs emphasize abnormalities of cholesterol and lipoprotein metabolism; and genetic or combined models are useful for dissecting the effects of specific genes or multifactorial lifestyle exposures on lipid metabolism.

### 4.2. Models of Diabetes and Its Complications

Diabetes comprises metabolic diseases characterized primarily by chronic hyperglycemia. Over time, hyperglycemia can cause multisystem complications affecting the retina, kidney, nervous system, foot, and cardiovascular system through oxidative stress, inflammation, endothelial injury, and related mechanisms [29,30]. Zebrafish models of diabetes and its complications are commonly established through high-glucose exposure, dietary induction, chemical induction, or genetic manipulation.

High-glucose exposure models generally place embryos, larvae, or adults in glucose-containing water to rapidly create a hyperglycemic environment and diabetes-related phenotypes. For example, zebrafish embryos exposed to high glucose develop enlarged retinal vessel diameters, providing a model for short-term hyperglycemia-induced retinal vascular changes and candidate-drug screening [31]. Similar glucose-immersion paradigms have been used by independent groups, which also reported persistent hyperglycemia and glucose-associated retinal, vascular, and neurological alterations, supporting the reproducibility of high-glucose zebrafish models across different experimental settings [32].

Diet-induced models more closely resemble metabolic dysfunction associated with type 2 diabetes. Ge et al. overfed 4-month-old male zebrafish for 4 weeks, inducing increased body weight, fasting blood glucose, serum triglycerides and cholesterol, and hepatic lipid-droplet accumulation. These features support use of the model to study overnutrition-induced glucose and lipid dysregulation and diabetes-like changes [33]. Consistent metabolic abnormalities have also been observed in independent diet-induced models, including obesity, impaired glucose tolerance, and insulin resistance, further supporting dietary manipulation as a reproducible approach for modeling type 2 diabetes-like metabolic dysfunction in zebrafish [34].

Chemically induced models commonly use streptozotocin (STZ) or alloxan to selectively damage pancreatic β cells, reduce insulin secretion, and induce hyperglycemia. They are widely used to model a type 1 diabetes-like state and to investigate diabetic complications and β-cell regeneration [35,36]. The NTR/MTZ β-cell-ablation strategy has subsequently been applied by independent groups to investigate β-cell regeneration, further supporting the reproducibility and utility of this targeted-ablation approach [37].

A complementary chemical–genetic approach is the nitroreductase/metronidazole (NTR/MTZ) system, in which *Escherichia coli* NAD(P)H-dependent nitroreductase NfsB (*nfsB*), a protein-coding gene encoding a nitroreductase enzyme, is specifically expressed in pancreatic β cells to enable MTZ-induced β-cell ablation and subsequent regeneration after MTZ withdrawal [38].

Genetic models of diabetes can be established by disrupting genes required for pancreatic development or β-cell function. Wiggenhauser et al. used pancreatic and duodenal homeobox 1 (*pdx1*)^−/−^ zebrafish, a loss-of-function model targeting a protein-coding gene that encodes a homeobox transcription factor required for pancreatic development and β-cell function, to establish a genetic diabetes model that displayed persistent hyperglycemia followed by proteinuria, damage to the glomerular filtration barrier, and tubular alterations resembling diabetic nephropathy. The model is therefore useful for studying genetically driven hyperglycemia and mechanisms of diabetic complications [6]. Other genetic approaches targeting glucose-homeostasis pathways have produced complementary diabetic phenotypes; for example, disruption of insulin-receptor signaling or glyoxalase 1 has resulted in impaired glucose regulation and hyperglycemia-associated metabolic abnormalities, illustrating convergent evidence from genetically distinct zebrafish models [39].

High-glucose exposure, dietary induction, chemical or chemical–genetic β-cell ablation, and genetic manipulation therefore provide complementary approaches for modeling different aspects of diabetes. Importantly, hyperglycemia, impaired glucose homeostasis, β-cell dysfunction, and diabetes-related tissue injury have been reproduced across multiple experimental strategies and independent studies, although the degree of independent validation varies among individual models.

### 4.3. Models of NAFLD/NASH

Nonalcoholic fatty liver disease (NAFLD) is a metabolic liver disease defined primarily by abnormal hepatic lipid accumulation, whereas nonalcoholic steatohepatitis (NASH) represents progression of NAFLD to inflammation, hepatocellular injury, and fibrosis [40].

Zebrafish models of NAFLD mainly include diet-induced and genetic steatosis models. Dietary models are suitable for overnutrition-associated NAFLD, whereas genetic models help dissect hepatic lipid deposition caused by abnormalities in specific genes or pathways. Li et al., for example, established an adult NAFLD model using a high-fat diet supplemented with egg-yolk powder. After 30 days of feeding, adult zebrafish showed increased BMI, serum triglycerides and total cholesterol, hepatic lipid droplets, and the liver-injury markers alanine aminotransferase (ALT) and aspartate aminotransferase (AST) [10]. Comparable hepatic steatosis and lipid-metabolic abnormalities have also been reported by independent groups using different high-fat or lipid-enriched dietary protocols, supporting the reproducibility of diet-induced zebrafish models of fatty liver disease [41]. In a genetic approach, Li et al. used ELOVL fatty acid elongase 2 (*elovl2*)-mutant zebrafish, a loss-of-function model targeting a protein-coding gene that encodes an endoplasmic-reticulum-localized fatty-acid elongase required for the biosynthesis of long-chain polyunsaturated fatty acids, including docosahexaenoic acid (DHA), and found increased hepatic lipid synthesis and uptake, reduced lipid oxidation and transport, lipid-droplet accumulation, NAFLD, and ferroptosis-mediated hepatocyte injury [4].

NASH models are established mainly through dietary induction, chemical exposure, or combined interventions. Wild-type zebrafish fed an HFD can develop hepatic steatosis, inflammation, and fibrosis; at the NASH stage, hepatic iron deposition, lipid peroxidation, and ferroptosis-related markers are also increased, making the model useful for studying NASH progression and oxidative injury [42]. Consistent liver phenotypes, including steatosis, inflammatory changes, and fibrosis-associated alterations, have been observed in independent diet-induced zebrafish studies, providing convergent support for the use of dietary models to investigate NAFLD/NASH progression [41]. Chemical induction is likewise used to create steatohepatitis-like injury. Ou-Yang et al. established a chemically induced NASH-like model using microcystin-LR (MC-LR): exposure to 10 μg/L MC-LR induced hepatic steatosis and toxic saturated-fatty-acid accumulation through a PPARα-related lipid metabolic mechanism, while accumulated saturated fatty acids, particularly palmitic acid, activated NOD1-associated inflammatory signaling, thereby promoting hepatic inflammation and NASH-like liver injury [43]. Combined models can better represent the multiple-hit progression from NAFLD to NASH. Kulkarni et al. developed a two-hit zebrafish model in which an HFD first induced hepatic lipid deposition, followed by liver-specific inflammation using the fatty acid-binding protein 10 (*fabp10*)-Cre/Cre-enabled tetracycline-inducible pro-inflammatory cytokines 3 (CETI-PIC3) system, in which doxycycline induces hepatocyte-specific expression of TNFα, IL-1β, and IFNγ. The second hit exacerbated hepatic inflammation, immune-cell infiltration, and stress responses [44]. By experimentally separating lipid deposition from inflammatory amplification, this model is particularly suitable for studying inflammation-driven mechanisms in early NASH.

Dietary induction, genetic manipulation, chemical exposure, and combined interventions thus reproduce different components of NAFLD/NASH through overnutrition, specific defects in lipid-metabolic pathways, toxic injury, and inflammatory amplification, respectively. Among these approaches, diet-induced hepatic steatosis and inflammatory liver phenotypes have been reproduced across multiple independent studies, whereas some genetic, chemical, and combined models currently serve as more pathway- or mechanism-specific experimental paradigms.

### 4.4. Models of Atherosclerosis

Atherosclerosis is a lipid-driven chronic inflammatory disease of the arteries characterized by endothelial dysfunction, lipid deposition, macrophage recruitment, and plaque formation. Plaque progression or rupture can lead to major cardiovascular events, including coronary heart disease, stroke, and peripheral arterial disease [45,46]. Zebrafish models of atherosclerosis are established mainly by HCD feeding, genetic manipulation, or combined inflammatory induction.

HCDs are commonly used to establish models of early atherosclerosis-like changes. Deng et al. fed 5-dpf larvae a 5% HCD for 10 days, inducing lipid accumulation, inflammation, and vascular endothelial injury [47]. Consistent findings have been reported by independent groups. Zheng et al. showed that high-cholesterol feeding increased total cholesterol and triglyceride levels and induced vascular lipid deposition and obstruction in zebrafish [48]. Zhang et al. reported that prolonged HCD feeding increased TC, TG, and LDL-C levels and produced vascular cholesterol accumulation, neutrophil aggregation, macrophage infiltration, and plaque-like lesions [49].

Genetic models increase susceptibility to atherosclerosis mainly by disrupting genes involved in lipoprotein metabolism and cholesterol homeostasis. Hu et al. used CRISPR/Cas9 to knock out zebrafish apolipoprotein Eb (*apoeb*), a protein-coding gene encoding a lipid-transport apolipoprotein involved in cholesterol efflux and triglyceride-rich lipoprotein clearance, and combined the mutation with short-term HFD feeding to establish an *apoeb*-mutant model of dyslipidemia. The model showed pronounced vascular lipid deposition, elevated whole-body and plasma lipid levels, and recruitment of neutrophils and macrophages, all of which are associated with early atherosclerosis [2]. Independent support for this genetic strategy has recently been provided by Wang et al., who established stable CRISPR/Cas9-generated *apoeb* knockout zebrafish and validated atherosclerosis-related phenotypes using lipid deposition, immune-cell, and vascular readouts [50]. These findings strengthen the evidence that *apoeb* deficiency reproducibly produces dyslipidemic and vascular phenotypes relevant to early atherosclerosis.

To enhance vascular inflammation and facilitate drug screening, some studies have combined an HCD with lipopolysaccharide (LPS) to induce early atherosclerosis-like changes. Han et al. treated zebrafish with HCD, LPS, or HCD plus LPS and observed cholesterol accumulation, vascular inflammation, disrupted lipid metabolism, and oxidative stress; they also evaluated the effects of atorvastatin, aspirin, and vitamin C [51]. This rapid model produces readily observable phenotypes and is useful for primary drug screening, but it is not equivalent to advanced, complex human plaques. Compared with HCD feeding and *apoeb*-based genetic models, however, the HCD-plus-LPS strategy has been less extensively validated across independent studies, and its reproducibility therefore requires further assessment.

In summary, convergent evidence from independent studies most consistently supports HCD feeding and apoeb deficiency for reproducing hyperlipidemia, vascular lipid deposition, and early inflammatory changes associated with atherosclerosis. In contrast, independent validation of accelerated inflammatory approaches such as HCD plus LPS remains more limited. Existing models still remain limited in their ability to model mature plaques and plaque rupture.

### 4.5. Models of Inherited Metabolic Diseases

Inherited metabolic diseases, also termed inborn errors of metabolism, are genetic disorders caused by variants in metabolism-related genes. Dysfunction of enzymes, transporters, or cofactors can cause abnormal substrate accumulation, product deficiency, or impaired energy metabolism and may affect amino-acid, organic-acid, fatty-acid, carbohydrate, mitochondrial, lysosomal, and other metabolic pathways [52]. Zebrafish models of inherited metabolic disease are established primarily through genetic manipulation, including transient knockdown, gene knockout, and modeling of patient variants.

Early functional studies in zebrafish frequently used morpholino oligonucleotides (MOs) to transiently knock down target genes and rapidly assess embryonic or larval phenotypes. MO knockdowns and stable mutants can display different phenotypes because of genetic compensation and related mechanisms; MO results are therefore best treated as preliminary leads and should be validated using stable mutants and independent evidence [53]. Gene-editing platforms such as CRISPR/Cas9, TALENs, and zinc-finger nucleases (ZFNs) can generate stable genetic models for subsequent studies of disease progression and treatment response [53].

Stable gene-deficient models are useful for studying inherited metabolic diseases. Loss of methylmalonyl-CoA mutase (*mmut*), a protein-coding gene that encodes a mitochondrial vitamin B12-dependent enzyme that converts methylmalonyl-CoA to succinyl-CoA, causes accumulation of methylmalonic acid and related metabolites and is associated with mitochondrial dysfunction, oxidative stress, and multiorgan injury; a stable CRISPR/Cas9 *mmut*-deficient zebrafish model reproduced methylmalonic acid accumulation, mitochondrial dysfunction, oxidative stress, impaired locomotor activity, and reduced survival, while liver-specific *mmut* re-expression further supported the specificity of these phenotypes [54]. Independent evidence from another inherited cobalamin disorder further supports this modeling strategy: *mmachc*-deficient zebrafish reproduced characteristic biochemical and developmental abnormalities of cobalamin C deficiency, including methylmalonic acid accumulation, growth impairment, retinal abnormalities, and early mortality [55]. In addition, zebrafish with loss of function of dihydrolipoamide dehydrogenase (*dldh*), a protein-coding gene that encodes a mitochondrial flavoprotein serving as the shared E3 subunit of several dehydrogenase complexes, model the mitochondrial metabolic disorder DLD deficiency and exhibit reduced locomotor performance, hepatic abnormalities, altered lactate/pyruvate metabolism, and disrupted branched-chain amino-acid metabolism, supporting mechanistic and therapeutic studies [3]. Together, these independently developed genetic models show that stable gene disruption can reproduce biochemical, organ-level, and functional abnormalities across distinct inherited metabolic diseases.

Patient-derived pathogenic variants can also be evaluated in zebrafish through complementation with human alleles or transgenic strategies, but expression of a patient variant must be distinguished carefully from knock-in at the orthologous zebrafish locus. Fan et al., for example, first transiently expressed RNA carrying the N370S variant of human glucosylceramidase beta 1 (*GBA1*), a protein-coding gene that encodes the lysosomal hydrolase glucocerebrosidase, in a zebrafish *gba1*-deficient background and subsequently generated a stable transgenic line with macrophage-specific expression of the human allele. The N370S allele failed to rescue macrophage lysosomal storage or resistance to mycobacterial infection, showing that this strategy can compare the function of specific human alleles [56]. Because N370S was not introduced precisely at the orthologous zebrafish *gba1* locus, interpretation should be limited to the function and cell-specific effects of the human variant in a *gba1*-deficient background. However, independent replication of individual patient-variant modeling strategies remains comparatively limited, and allele-specific conclusions should therefore be interpreted cautiously.

In inherited metabolic disease research, transient knockdown is suitable for rapidly identifying clues to gene function, stable knockout or loss-of-function models can reveal long-term metabolic abnormalities, and complementation, transgenic expression, or precise knock-in of patient variants can help assess the function of specific pathogenic alleles.

## 5. Phenotypic Validation of Zebrafish Models of Metabolic Disease

### 5.1. Morphological Validation: Assessing Tissue and Organ Lesions

Morphological validation is the most fundamental step in phenotyping zebrafish models of metabolic disease and is used primarily to determine whether disease-related structural changes have developed in tissues and organs.

Morphological validation is generally performed at the organ, tissue, and cellular levels. First, the imaging method should match the target organ: hepatic lipid droplets can be assessed by live imaging in larvae [9]; vascular lipid deposition and immune-cell recruitment can be visualized using fluorescent lines and confocal microscopy [2]; and renal and retinal structures can be examined using relevant nephropathy models and retinal vascular imaging methods, respectively [57,58]. Second, tissue sections and histological stains can reveal hepatocellular steatosis, tissue disorganization, inflammatory-cell infiltration, or fibrosis [10,59]. Oil Red O, Nile Red, BODIPY, and LipidTOX can be used to visualize lipid droplets or neutral-lipid accumulation [9]. Finally, electron microscopy can reveal ultrastructural changes in structures such as the glomerular filtration barrier and mitochondria [3,58].

Overall, morphological validation provides direct evidence of tissue- or organ-level pathology and is an important indicator of successful modeling. It should nevertheless be complemented by functional measures of blood glucose, circulating lipids, liver function, inflammation, oxidative stress, and gene expression to provide a more complete evaluation of zebrafish metabolic-disease models.

### 5.2. Functional Validation: Detecting Metabolic Dysfunction and Associated Injury

Morphology can show whether organ structure has changed, but it cannot fully establish the presence of disturbed glucose or lipid metabolism, hepatic or renal dysfunction, inflammation, or oxidative stress. Functional endpoints are therefore required to confirm the successful establishment of a zebrafish metabolic-disease model.

Measures of glucose and lipid metabolism are central to functional validation. Diabetes models can be assessed using blood glucose, glucose uptake, glycogen content, and insulin-signaling endpoints to evaluate hyperglycemia, abnormal glucose metabolism, and insulin resistance [60,61]. Obesity and fatty-liver models can be evaluated using triglycerides, total cholesterol, and other lipid measures [10,21]. Atherosclerosis-related models such as *apoeb* mutants can also combine whole-body and plasma lipid quantification with vascular imaging to assess lipid deposition [2].

Measures of organ injury, inflammation, and oxidative stress are also important. In NAFLD/NASH and other liver-injury models, ALT and AST can be used to evaluate hepatocellular injury [10]. Models of diabetic nephropathy or metabolic kidney injury can be assessed using proteinuria, renal-function measures, or kidney-injury markers [58].

Functional validation therefore establishes whether metabolic dysfunction and associated injury have actually occurred and is essential for judging model reliability. Because individual larvae are small, some biochemical assays require pooled samples, which may obscure interindividual variation.

### 5.3. In Vivo Observation: Tracking Disease Dynamics

In vivo observation is a major strength of zebrafish models of metabolic disease. The transparency of embryos and larvae permits direct visualization of changes in the liver, vasculature, pancreas, immune cells, and lipid deposition without disrupting the organism. In addition, pigmentation-deficient lines such as *casper* and *crystal* retain high optical transparency into adulthood, extending the applicability of in vivo imaging to adult zebrafish [62,63].

Fluorescent reporter lines and live-imaging techniques can be used to observe a range of metabolic-disease phenotypes. For hepatic steatosis, the hepatocyte-specific Tg(fabp10a:EGFP-PLIN2) reporter labels hepatic lipid droplets through EGFP-tagged perilipin 2 (PLIN2), enabling direct visualization and quantification of lipid-droplet number, size, and dynamics by live fluorescence imaging [64]. Hepatocyte-specific Tg(fabp10a:mCherry) and Tg(fabp10a:GFP) lines can additionally delineate the liver and permit longitudinal assessment of changes in liver area during drug-induced liver injury and regeneration [65]; retinal vascular imaging can assess hyperglycemia-induced changes in vessel diameter and vascular-cell morphology [31,57]; and vascular imaging in *apoeb* mutants can reveal lipid deposition and the recruitment of neutrophils and macrophages [2]. Repeated observation of the same individual or cohort provides dynamic information on disease onset, progression, and treatment response.

Compared with static validation and endpoint assays, live imaging emphasizes dynamic processes, links static phenotypes to disease progression, and provides intuitive evidence for subsequent drug screening and efficacy evaluation.

### 5.4. Mechanistic Clues from Omics and Single-Cell Analyses

Transcriptomics, metabolomics, and proteomics characterize molecular alterations in zebrafish metabolic-disease models at the levels of gene expression, metabolites, and proteins and can identify changes in lipid metabolism, insulin signaling, inflammation, oxidative stress, mitochondrial function, and related pathways [66]. Single-cell transcriptomics can further localize global molecular changes to specific cell populations, including hepatocytes and immune cells [67,68]. These methods help determine whether phenotypes are accompanied by disease-consistent pathway changes and support the discovery of potential biomarkers and therapeutic targets.

Single-cell RNA sequencing can distinguish cell populations such as hepatocytes, endothelial cells, macrophages, and hepatic stellate cells, thereby resolving the cellular origins of metabolic stress, inflammation, and fibrosis [68]. The primary role of omics and single-cell analyses is therefore not to prove model validity on their own, but to generate leads for mechanistic studies and drug-target discovery.

### 5.5. High-Throughput Quantification: Automated Imaging and AI Analysis

As zebrafish are increasingly used for metabolic-disease drug screening, manual observation and quantification are no longer sufficient for large-scale experiments. Automated imaging and AI-assisted analysis can increase the efficiency of phenotype acquisition and data processing.

Zebrafish embryos and larvae can be cultured and treated in 96- or 384-well plates, enabling automated microscopy systems to rapidly acquire large numbers of phenotypic images [69]. Standardized image-analysis pipelines can then extract quantitative measures such as vessel length, branch number, fluorescence intensity, and lesion area. For example, the ZVQ tool automatically quantifies the three-dimensional cerebrovascular structure of zebrafish [70]. Automated acquisition and analysis improve the consistency and efficiency of large-scale screening.

Overall, high-throughput quantitative analysis enables faster and more objective evaluation of zebrafish phenotypes. It improves standardization of model validation and provides a technical basis for drug screening, efficacy evaluation, and prioritization of candidate compounds.

The representative modeling strategies, major phenotypes, appropriate applications, and key limitations of zebrafish models across metabolic-disease categories are summarized in Table 1.

## 6. Mechanistic Investigation

**Figure 2:**
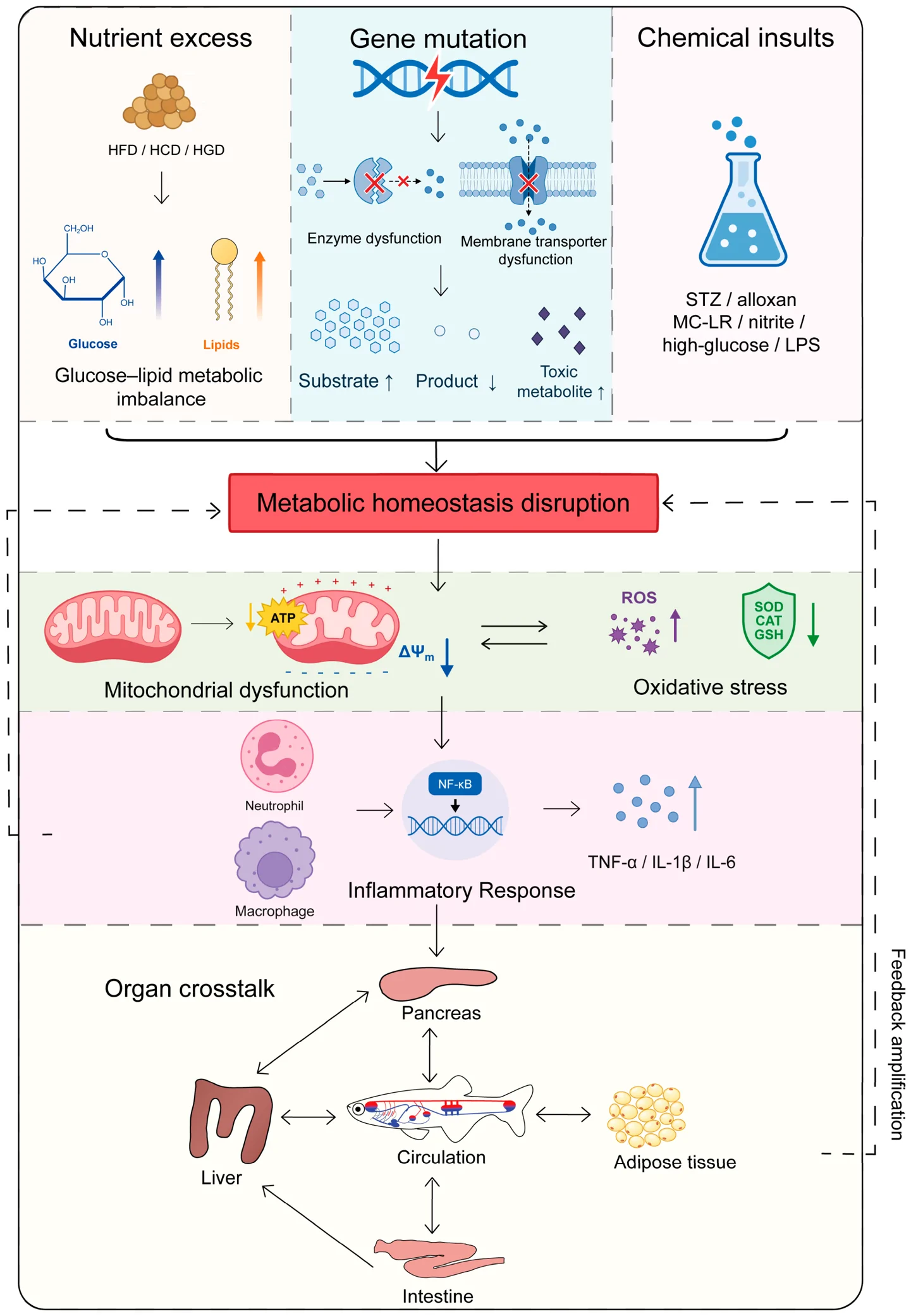
Integrated mechanisms of metabolic homeostasis disruption in zebrafish metabolic disease models. The zebrafish liver schematic was adapted from Figure 2 of Mo et al. [117]; the zebrafish intestinal schematic from Figure 1B of Xia et al. [118]; the neutrophil illustration from Figure 1 of Parker et al. [119]; and the zebrafish vascular system from Figure 2 of Hoareau et al. [18]. All four source articles are distributed under the Creative Commons Attribution 4.0 International (CC BY 4.0) license. Background colors denote the four mechanistic stages (triggers, mitochondrial/oxidative stress, inflammation, organ crosstalk). Red × marks indicate loss of enzyme/transporter function; ↑/↓ indicate increases/decreases; solid arrows show the pathological cascade, and dashed arrows/brackets show the feedback-amplification loop.

Although different zebrafish models may converge on common pathological processes such as dysregulated glucose and lipid metabolism, mitochondrial injury, oxidative stress, and inflammation, differences in inducing factors, developmental stage, and intervention targets mean that the mechanistic level and strength of causal inference vary among models. This section therefore not only summarizes these shared mechanisms but also compares the distinct value and evidentiary limits of dietary, chemical, and genetic models for mechanistic investigation. Representative model-specific procedures and phenotypes are summarized in Table 1; the discussion below focuses on differences in mechanistic interpretation and causal inference (Figure 2).

### 6.1. Disruption of Metabolic Homeostasis and Its Major Triggers

Although metabolic diseases have diverse specific etiologies, most ultimately involve disruption of metabolic homeostasis. Nutrient excess, genetic defects, and chemical or other exogenous stressors can induce metabolic abnormalities by increasing the glucose and lipid load, blocking specific metabolic pathways, or directly injuring cells and organs. Different triggers may ultimately converge on mitochondrial dysfunction, oxidative stress, and inflammation, but their initiating mechanisms and the scope of interpretation they support are not equivalent.

Chronic exposure to high levels of glucose or fat and sustained nutrient excess are major drivers of common metabolic diseases, including obesity, type 2 diabetes, fatty liver disease, and metabolic syndrome [83,84,85]. Prolonged overconsumption disrupts glucose and lipid homeostasis, resulting in abnormal blood glucose and lipid levels, increased hepatic lipogenesis, and tissue lipid deposition [86,87]. These abnormalities increase cellular metabolic load and may be accompanied by mitochondrial dysfunction, enhanced oxidative stress, and activation of inflammatory responses, thereby promoting tissue injury and disease progression [88,89].

Inherited metabolic diseases arise mainly from dysfunction of metabolic enzymes, transporters, or other metabolic regulators. The resulting blockage of specific pathways can cause substrate accumulation, deficiency of downstream products, and, in some cases, accumulation of toxic metabolites, ultimately impairing cellular and organ function [90,91].

Chemical or other exogenous stressors generally disrupt metabolic homeostasis by directly injuring specific cells or organs or by rapidly inducing oxidative stress and inflammation. LPS can directly activate reactive oxygen species (ROS) production and NF-κB-associated inflammatory pathways. Because it enhances inflammation when combined with dietary factors, metabolic inflammation must be distinguished carefully from the direct inflammatory effects of LPS in such composite models [51,92].

Different zebrafish modeling strategies illuminate distinct aspects of glucose and lipid metabolism. High-fat or high-glucose dietary models primarily simulate chronic nutrient excess and permit observation of the continuum from impaired insulin signaling to increased lipogenesis and tissue lipid deposition. Because many metabolic pathways change simultaneously, however, they are less suited to assigning causality to a single molecule [34,93]. Chemical models such as glucose immersion act rapidly and are more suitable for studying early metabolic responses to hyperglycemic exposure and glucotoxicity, but they generally reproduce systemic obesity and chronic disease less completely than dietary models [21,94]. By contrast, genetic models involving *apoeb*, miR-7a, or other defined factors can localize specific molecular steps in lipoprotein transport or regulation of lipid synthesis and therefore offer clearer causal direction, although they do not fully represent common metabolic diseases driven by nutrient excess [2,95]. Dietary models are consequently better suited to studying the development and progression of metabolic abnormalities, whereas genetic models are particularly valuable for validating specific regulatory pathways.

Thus, although nutrient-excess and genetic models may both show disruption of metabolic homeostasis, the former primarily reflect increased glucose and lipid burden, whereas the latter emphasize obstruction of specific metabolic pathways. Their mechanistic interpretations are not interchangeable.

### 6.2. Mitochondrial Dysfunction and Oxidative Stress

After glucose and lipid metabolism or another metabolic pathway becomes dysregulated, cells must process excessive glucose, fatty acids, or abnormally accumulated metabolites, increasing the metabolic burden on mitochondria [96,97]. Sustained metabolic stress can damage mitochondrial structure and function, leading to reduced ATP production, impaired electron-transport-chain efficiency, and increased generation of ROS [98]. Excessive ROS disrupt redox homeostasis and causes oxidative stress [99].

The initiating point of mitochondrial injury differs among zebrafish models. Nitrite-induced models primarily show ROS accumulation, loss of mitochondrial membrane potential, and reduced ATP production caused by exogenous chemical stress. They are useful for studying the direct relationship between oxidative injury and mitochondrial dysfunction, although their inducing factor does not closely match that of common chronic human metabolic diseases [100]. Adult HFD models begin with sustained nutrient excess and show mitochondrial fragmentation and reduced mitochondrial biogenesis, fatty-acid oxidation, and oxidative phosphorylation, more closely resembling the course of chronic metabolic diseases such as NAFLD [59]. The former is advantageous for identifying acute oxidative-stress processes, whereas the latter is more suitable for evaluating failed mitochondrial adaptation under chronic metabolic pressure; the multiple pathways altered by an HFD, however, complicate causal attribution.

Mitochondrial dysfunction and oxidative stress can therefore be viewed as a mechanistic link between disrupted metabolic homeostasis and tissue injury. Metabolic abnormalities increase mitochondrial burden and promote ROS accumulation, while sustained oxidative stress further aggravates mitochondrial damage, creating a vicious cycle that facilitates subsequent inflammation and organ dysfunction.

### 6.3. Inflammation and Immunometabolism

Inflammation is an important amplifier of metabolic-disease progression in zebrafish. Disrupted glucose and lipid metabolism, mitochondrial dysfunction, increased ROS, and lipid peroxidation place cells in an injured state [101]. Damaged cells can release abnormal metabolic and damage-associated signals that recruit and activate macrophages, neutrophils, and other immune cells. Inflammatory mediators and pathways, including TNF-α, IL-1β, IL-6, MCP-1, and NF-κB, are subsequently upregulated, producing chronic low-grade inflammation [102,103].

Under physiological conditions, immune cells obtain energy through glycolysis, oxidative phosphorylation, and fatty-acid oxidation. High glucose, high lipid levels, oxidative stress, and inflammatory environments alter these metabolic processes, thereby affecting immune-cell activation, migration, and cytokine secretion [104,105]. Metabolic dysfunction therefore not only induces inflammation but also reprograms immune-cell metabolism in a manner that favors persistence of the inflammatory response.

Zebrafish models can reveal links between metabolic abnormalities and inflammation. For example, apoeb mutation combined with short-term HFD feeding elevates whole-body and plasma lipid levels, promotes vascular lipid deposition, and recruits neutrophils and macrophages, suggesting that disturbed lipoprotein metabolism promotes early vascular inflammation [2].

The range of inflammatory mechanisms that can be inferred also differs among models. High-fat or high-cholesterol diets reveal relationships among lipid load, tissue injury, and recruitment of macrophages or neutrophils and are therefore suitable for investigating the gradual transition from metabolic dysfunction to chronic low-grade inflammation [106]. Combining LPS or another inflammatory stimulus with dietary factors can enhance the inflammatory phenotype and shorten the modeling period. Because the exogenous stimulus itself activates NF-κB and related cytokines, however, such findings cannot independently establish that the inflammation was caused entirely by metabolic dysfunction [51,92]. Fluorescent immune-cell reporter lines or pathway-specific genetic manipulations can be added to dynamically track immune-cell migration and verify the role of particular signals [107]. Dietary models are thus better suited to the natural development of metabolic inflammation, composite models to rapid assessment of anti-inflammatory interventions, and genetic or reporter lines to dissection of cellular and molecular mechanisms.

Inflammation and immunometabolism therefore constitute a key step by which metabolic abnormalities expand into tissue injury. Disrupted metabolic homeostasis and cellular damage activate inflammation, while persistent inflammation feeds back to worsen insulin resistance, lipid deposition, oxidative stress, and organ dysfunction, further driving disease progression.

### 6.4. Interorgan Metabolic Crosstalk

Metabolic diseases are generally systemic disorders involving multiple organs rather than lesions confined to a single organ. Dysregulated glucose and lipid metabolism, mitochondrial injury, and inflammation may begin locally in the liver, pancreas, adipose tissue, intestine, or vasculature. As disease progresses, these local changes affect other organs through hormones, inflammatory mediators, lipid metabolites, bile acids, microbiota-derived metabolites, and related signals, ultimately producing systemic metabolic dysfunction [108,109].

Interactions among the zebrafish liver, pancreas, adipose tissue, intestine, and vasculature are particularly important in this process. After pancreatic β-cell ablation, glucose- and lipid-metabolic processes change in hepatocytes, while the hepatocyte molybdenum-cofactor biosynthesis pathway contributes to β-cell regeneration, illustrating bidirectional liver-pancreas communication [110]. The intestinal microbiota can promote fatty-acid absorption and lipid-droplet formation in the intestinal epithelium and liver. Activation of Lxr in intestinal epithelial cells can also temporarily store dietary lipid, delay its entry into the systemic circulation, and attenuate HFD-induced hypercholesterolemia and hepatic steatosis, demonstrating metabolic communication along the intestine-liver and intestine-circulation axes [111,112]. Vegfa signaling from β cells contributes to islet vascularization, whereas disruption of pancreatic blood vessels leads to disorganized islet architecture, reduced islet function, and impaired glucose regulation, indicating bidirectional interactions between the pancreas and its local vasculature [113,114]. Endothelial signaling in the zebrafish liver regulates hepatocyte polarity, hepatic vascular and biliary networks, and metabolic zonation, showing that vascular endothelium not only transports metabolites but also directly regulates hepatic structure and metabolic function [115,116].

Interorgan metabolic crosstalk is therefore essential to understanding the complex phenotypes of metabolic disease. Dysregulated glucose and lipid metabolism, mitochondrial dysfunction, oxidative stress, and inflammation do not occur in isolation; through multiorgan signaling, they jointly drive systemic metabolic dysfunction and the progression of associated complications (Figure 2).

## 7. Applications of Zebrafish Metabolic-Disease Models in Therapeutic Research

### 7.1. Therapeutic Applications Across Metabolic-Disease Models

Intervention studies in models of obesity and dyslipidemia focus mainly on inhibiting adipogenesis, enhancing fatty-acid β-oxidation pathways, increasing energy expenditure, and improving circulating lipid profiles. Overfeeding or HFD feeding induces weight gain, visceral fat accumulation, and dyslipidemia in zebrafish and is suitable for evaluating the integrated effects of lipid-lowering agents and natural bioactive compounds [17,120,121]. In addition to body weight and length, efficacy endpoints should include visceral adipose volume, plasma triglycerides and total cholesterol, hepatic lipid deposition, and energy-metabolism pathways, thereby distinguishing simple weight loss from genuine improvement in lipid metabolism [122,123].

Diabetes models can be used to screen drugs that lower glucose, improve insulin resistance, protect β cells, or promote β-cell regeneration. High-glucose exposure and diet-induced models mainly represent disturbed glucose homeostasis and insulin resistance, whereas β-cell injury or ablation models are more suitable for evaluating β-cell-protective and regenerative agents [124,125,126,127]. Relevant efficacy endpoints should include blood glucose, tissue glucose uptake, insulin expression, β-cell number, and functional responses to glucose stimulation [128,129]. For diabetic complications, organ-specific endpoints for the retina, kidney, nervous system, or vasculature should also be included [58,130,131,132].

In NAFLD models characterized predominantly by simple hepatic steatosis, efficacy assessment should focus on hepatic lipid deposition and may incorporate measures of lipogenesis, fatty-acid oxidation, and mitochondrial function to analyze drug action [44,59,133]. When a model additionally develops NASH features such as inflammation, oxidative stress, and hepatocellular injury, reduced hepatic lipid staining alone is not sufficient evidence of efficacy; inflammatory mediators, ROS, and liver-tissue injury should also be assessed. If fibrosis is present, deposition of collagen and other extracellular-matrix components should be measured [134,135,136].

Atherosclerosis models are used mainly to evaluate lipid-lowering, anti-inflammatory, antioxidant, and vasoprotective agents. Transparent zebrafish and fluorescent transgenic lines allow continuous in vivo observation of drug effects on vascular lipid deposition, inflammatory-cell recruitment, and plaque-like lesions [2,80]. Because these models generally represent early vascular lesions, their principal value lies in candidate-drug screening and mechanistic studies; mammalian models remain necessary to predict effects on mature plaque stability and cardiovascular events [2,137,138].

Models of inherited metabolic disease have defined pathogenic genes and metabolic defects and can be used to evaluate metabolite supplementation, substrate reduction, maintenance of mitochondrial DNA, and improvement of respiratory-chain function. Efficacy endpoints should correspond to the specific disease mechanism and may include abnormal metabolite levels, mitochondrial DNA copy number, oxygen consumption, locomotor performance, growth and development, and survival [139,140]. Because different mutant models may respond differently to the same drug, zebrafish can also be used to examine the relationship between therapeutic response and genetic background.

Representative therapeutic studies are summarized in Supplementary Table S1 [141,142,143,144,145,146,147,148,149,150,151,152,153,154,155,156,157,158,159,160,161,162,163,164].

### 7.2. Reliability and Translation of Efficacy Evaluation

The reliability of efficacy results in zebrafish depends first on whether the selected disease model reflects the disease stage and key pathological phenotype targeted by the therapy. Short-term diet-induced models usually represent early metabolic abnormalities and are suitable for evaluating agents intended to improve lipid or glucose metabolism [44]. Models that also display inflammation, tissue injury, or fibrosis are more appropriate for evaluating anti-inflammatory, tissue-protective, or antifibrotic therapies [135,136]. The disease stage represented by a model should therefore be stated explicitly, and efficacy endpoints should be selected according to the intended drug action. Normal, disease-model, vehicle, and positive-control groups should also be included.

Second, a drug should show a reproducible dose–response relationship, and nonspecific toxicity must be excluded. Drug concentration in the water is not equivalent to actual exposure within the fish; solubility, stability, and absorption efficiency can all affect experimental results [165,166]. Multiple concentrations should therefore be tested to determine the minimum effective dose and toxic range, while survival, developmental status, heart rate, and locomotor activity are monitored simultaneously. Feeding behavior can also be assessed in adults or larvae that have begun exogenous feeding [167,168,169]. Efficacy is credible only when stable improvement in the disease phenotype occurs within a dose range that causes no evident toxicity [170,171].

Efficacy conclusions should also be supported jointly by phenotypic, biochemical, histological, and molecular endpoints. A single change in lipid fluorescence, blood glucose, or gene expression is useful for primary screening but is insufficient by itself to demonstrate disease improvement [129,133]. Concordant changes across multiple levels reduce the risk of false interpretation caused by measurement error or nonspecific effects. At the same time, drug mechanisms should be established causally through receptor antagonism, target-gene knockdown, gene knockout, or phenotypic rescue rather than inferred solely from altered expression of an associated pathway [136,172].

Cross-model validation is an important step in assessing translational potential. A reasonable workflow begins with high-throughput screening and early safety assessment in larvae, proceeds to validation in adults or a second zebrafish disease model, and then advances to mammalian models and human-derived cells or organoids [7,129]. Concordant cross-species findings increase confidence in efficacy, whereas discrepancies may reveal species differences in absorption, metabolism, drug targets, or disease mechanisms [7,129,173].

Zebrafish and humans differ in drug absorption, distribution, hepatic metabolic enzymes, clearance, and aspects of lipoprotein regulation. An effective concentration in zebrafish water or an internal exposure level therefore cannot be converted directly into a human therapeutic dose by simple proportional scaling [166,173,174]. A positive zebrafish result should be interpreted as evidence that a candidate has in vivo activity and merits further study, not as direct proof of clinical efficacy. A candidate can be considered to have high translational potential only when the model matches the therapeutic target, efficacy is dose-dependent, toxicity has been excluded, the mechanism has been validated, and findings are reproducible across models.

## 8. A Framework for Selecting Zebrafish Models of Metabolic Diseases

Selection of a zebrafish model of metabolic disease should begin with the research objective and the disease stage to be represented. Investigators should first determine whether the study concerns early phenotypic observation, candidate screening, mechanistic investigation, disease progression, complication assessment, or therapeutic validation. They should then decide whether the target phenotype is limited to metabolic dysfunction or includes organ lipid deposition, inflammation and tissue injury, fibrosis, or chronic complications [7,66,71]. Different objectives impose different requirements for model duration, pathological completeness, and depth of validation. Early screening, for example, focuses on whether a model rapidly and reproducibly generates a quantifiable phenotype, whereas studies of progression and treatment require corresponding histopathological and organ-functional changes.

The developmental stage and modeling strategy should then be selected according to the required depth of investigation. The relative strengths of larval and adult stages are discussed in Section 3, and representative model-specific applications and limitations are summarized in Table 1. When both screening efficiency and disease completeness are important, a two-stage larval rapid-screening-to-adult-systemic-validation strategy can be adopted.

Once a model has been selected, the minimum validation criteria required to support the proposed disease label and conclusions should be defined before the experiment begins. The validation approaches are described in Section 5, while disease-specific minimum criteria and limits of inference are summarized in Table 2.

**Figure 3:**
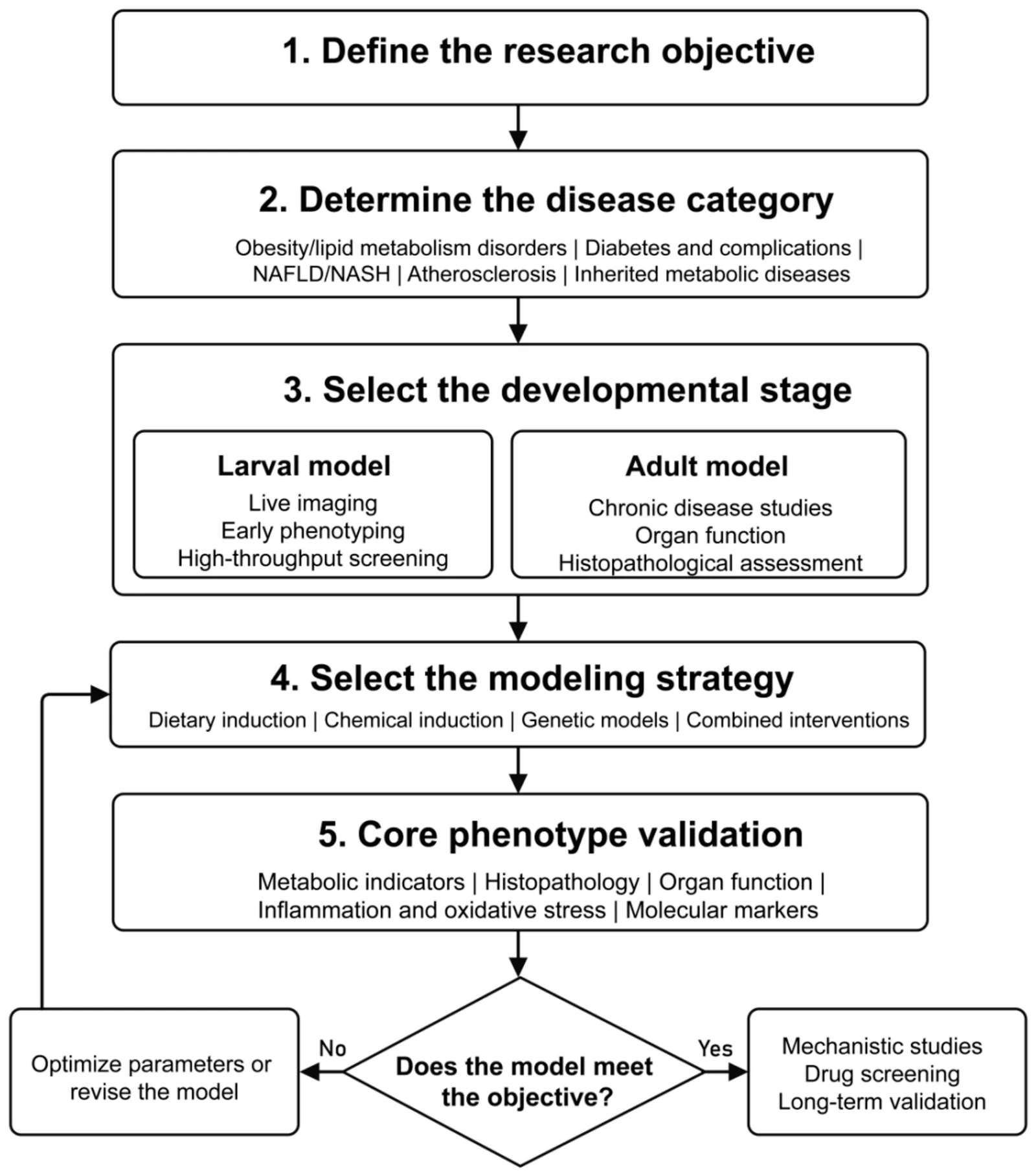
Decision-Making Framework for the Selection and Validation of Zebrafish Models of Metabolic Diseases.

The conclusions supported by a model should remain within the scope of the evidence actually obtained. If a model does not meet the predefined minimum validation criteria, the developmental stage, induction dose, treatment duration, or modeling method should be adjusted, or the disease designation and scope of inference should be narrowed accordingly [71]. Only after the predefined criteria have been met should the model advance to mechanistic analysis, efficacy evaluation, and cross-model translational validation [7,71]. Figure 3 summarizes this selection and adjustment process: investigators may first use Figure 3 to identify the overall pathway and then consult Table 1 to select a specific model.

As a practical example, the framework in Figure 3 can be applied to the high-carbohydrate diet (HCD)-induced obesity model reported by Virote et al. [175]. First, the research objective is defined as investigating the metabolic and adipose-tissue consequences of chronic nutritional excess. Second, this objective places the study within the obesity/lipid metabolism disorders category. Third, an adult model is selected because chronic metabolic alterations and adipose-tissue changes are the principal outcomes of interest. Fourth, dietary induction is chosen as the modeling strategy, with a high-carbohydrate diet used to reproduce nutrition-associated obesity. Fifth, core phenotype validation includes increased body weight, visceral and subcutaneous adipocyte hypertrophy, and elevated plasma glucose and triglyceride levels. Because these endpoints support the predefined research objective, the model can proceed to mechanistic studies or intervention testing. If the required phenotypes are not reproduced, the framework instead directs investigators to optimize the dietary parameters or revise the modeling strategy before proceeding.

## 9. Limitations and Future Perspectives

Despite the rapid development, high fecundity, convenient genetic manipulation, and suitability for in vivo imaging and large-scale screening of zebrafish, their use in metabolic-disease research has several limitations. First, zebrafish differ from mammals in lipid metabolism, adipose-tissue characteristics, endocrine regulation, and drug metabolism; metabolic phenotypes and drug effects observed in zebrafish therefore cannot be equated directly with human disease processes [17,21]. Second, current models lack sufficient standardization of induction conditions and evaluation criteria [33,58,129,130,131,132]. Studies differ in zebrafish strain, developmental stage, sex, feed composition, feeding amount, exposure duration, and environmental conditions, potentially producing differences in growth, metabolic phenotypes, and drug responses and thereby impairing reproducibility and cross-study comparison [9,10,42].

Future research should first strengthen standardization of zebrafish metabolic-disease models by defining induction conditions, disease stage, core phenotypes, and minimum validation criteria and by establishing reproducible workflows for husbandry, imaging, sampling, and data analysis [42,135,136]. Cross-model and cross-species validation should also be expanded. Targets or candidate drugs with translational potential should be evaluated progressively in human-derived cells or organoids, mammalian models, and clinical samples as appropriate to the research stage, with comparison of direction of effect, dose–response relationships, and mechanistic consistency across models [2,80,106]. As automated imaging, AI-based phenotype recognition, multi-omics integration, and humanized models advance, zebrafish are expected to become an increasingly important platform connecting mechanistic investigation, candidate screening, and preclinical translational evaluation [3,53,82,140].

## Figures and Tables

**Table 1:** Representative Modeling Strategies, Applications, and Key Limitations of Zebrafish Models of Metabolic Diseases.

Disease Category	Zebrafish Stage	Modeling Method and Core Procedure	Major Phenotypes and Validation	Applications and Key Limitations	References
Obesity and dyslipidemia	Adult	Dietary induction: Artemia overfeeding (5 vs. 60 mg/day for 9 weeks) or HFD40 feeding for 8 weeks	Increased body weight/BMI, visceral adiposity, whole-body and hepatic/muscle lipids, and TG/TC; validated by lipid staining, circulating lipids, and histology	Models overnutrition-induced obesity and supports long-term intervention studies; lengthy protocol and sensitive to feed composition, actual intake, sex, and age	[17,19,21,71,72,73]
	Larval or adult	Dietary induction: high-cholesterol feed to induce hypercholesterolemia and disturbed lipoprotein metabolism	Increased ApoB-LPs, plasma cholesterol, and cholesteryl esters, sometimes with fasting-associated hepatic steatosis	Suitable for cholesterol and lipoprotein metabolism; not a general obesity or NASH model, and results depend on fasting status and diet composition	[22,40,74]
	Larval or adult	Genetic models: Dox-inducible *mxd3* overexpression; CRISPR/Cas9 knockout of *leap2*; *cobll1a* mutation	Increased food intake, body weight, or visceral adiposity; hepatic lipid droplets and systemic lipid accumulation; altered lipid synthesis, catabolism, or transport	Strong construct validity for specific genes and pathways; phenotypes are gene-specific and may be influenced by genetic compensation	[7,25,26,27,53]
	Adult	Combined intervention: HFD plus sleep deprivation to model unhealthy diet and circadian disruption	Exacerbated lipid deposition and hepatic steatosis, with inflammation and NF-κB activation	Suitable for multifactorial lifestyle exposure; sleep-deprivation intensity and stress responses may introduce confounding	[28,71,75]
Diabetes and complications	Embryonic, larval, or adult	High-glucose exposure: glucose in the water to create an acute hyperglycemic environment	Abnormal blood glucose or 2-NBDG uptake, impaired insulin signaling, retinal vascular changes, and increased ROS	Rapid model for glucotoxicity, vascular injury, and primary drug screening; mainly reflects acute exposure and does not fully reproduce chronic diabetes	[12,31,35,76]
	Adult	Dietary induction: overfeeding 4-month-old males with frozen bloodworms for 4 weeks	Increased body weight, fasting glucose, TG/TC, and hepatic lipid droplets, with molecular changes associated with insulin resistance	Relatively close to overnutrition-induced type 2 diabetes; requires control of sex, feed, intake, and fasting time, and usually lacks chronic complications	[12,33,35,71,72,73]
	Larval or adult	Chemical induction: STZ or alloxan delivered by immersion, oral administration, or injection to damage pancreatic β cells	Reduced β-cell number, decreased insulin secretion, and hyperglycemia, sometimes with delayed wound healing; validated by β-cell imaging, glucose, and regeneration endpoints	Suitable for β-cell injury, regeneration, and therapeutic response; chemical toxicity and delivery route may cause nonspecific injury	[12,35,36]
	Embryonic	Chemical–genetic ablation: β-cell-specific expression of *E. coli nfsB* combined with metronidazole (MTZ) treatment	Loss of insulin-positive β cells following MTZ treatment, followed by β-cell regeneration after MTZ withdrawal	Requires transgenic lines and primarily models acute targeted β-cell ablation, with limited representation of the complex pathophysiology of diabetes	[37,38,77]
	Adult	Genetic manipulation: disruption of *pdx1*, a gene involved in pancreatic development and β-cell function	Persistent hyperglycemia, proteinuria, and glomerular filtration-barrier and tubular injury; validated by glucose, proteinuria, renal histology, and electron microscopy	Suitable for chronic hyperglycemia and diabetic-nephropathy-like changes; developmental defects may confound adult metabolic phenotypes	[6,12,35]
NAFLD	Adult	Dietary induction: high-fat feed supplemented with egg-yolk powder for 30 days	Increased BMI, TG/TC, ALT/AST, and hepatic lipid droplets with steatosis; validated by histology and lipid staining	Suitable for overnutrition-associated NAFLD and efficacy assessment; short-term models have limited inflammation, fibrosis, and long-term progression	[10,40,74]
	Larval or adult	Genetic manipulation: *elovl2* deficiency causing impaired DHA biosynthesis	Increased lipid synthesis/uptake, reduced oxidation/transport, hepatic lipid droplets, lipid peroxidation, and ferroptosis-related injury	Suitable for DHA deficiency and lipid-oxidation mechanisms; a single-gene model that does not represent most multifactorial NAFLD	[4,7,74]
NASH	Adult	Dietary induction: sustained HFD feeding in wild-type zebrafish	Hepatic steatosis, inflammation, and fibrosis; at the NASH stage, increased hepatic iron, lipid peroxidation, and ferroptosis-related markers; validated histologically and molecularly	Suitable for NAFLD-to-NASH/fibrosis progression and ferroptosis; severity varies with diet, duration, and individual differences, and NASH requires histological confirmation rather than HFD exposure alone	[11,21,42,78,79]
	Larval or adult	Chemical induction: exposure to 10 μg/L microcystin-LR	Hepatic steatosis, saturated-fatty-acid accumulation, and PPARα-NOD1-associated inflammation; validated by histological, lipid, and inflammatory endpoints	Suitable for toxicant-induced NASH-like mechanisms; etiology differs from human overnutrition-associated NASH, requiring cautious extrapolation	[43,74]
	Adult	Combined intervention: HFD-induced lipid deposition followed by liver-specific inflammation in fabp10-CETI-PIC3 fish	Enhanced hepatic lipid deposition, immune-cell infiltration, and stress responses, producing early NASH-like progression	Separates lipid deposition from inflammatory amplification; inflammation is artificial, and late fibrosis and natural history remain incompletely modeled	[7,43,44,74]
Atherosclerosis	Larval	Dietary induction: high-cholesterol feed to induce vascular lipid deposition and lipoprotein oxidation	Increased cholesterol and vascular lipid deposition, with macrophage/neutrophil recruitment and oxidative stress	Suitable for live imaging and primary screening of antiatherosclerotic drugs; models early lesions rather than mature plaques or plaque rupture	[47,80]
	Larval and adult	Genetic manipulation: CRISPR/Cas9 knockout of *apoeb* combined with short-term HFD feeding	Elevated whole-body and plasma lipids, vascular lipid deposition, and neutrophil and macrophage recruitment	Suitable for ApoE-related lipoprotein metabolism and vascular inflammation; inherited hyperlipidemia does not represent all sporadic disease	[2,7,80]
	Larval	Combined induction: LPS added to HCD to enhance inflammation and oxidative stress	Increased vascular lipid deposition, inflammatory-cell adhesion, TNF-α/IL-1β/IL-6, NF-κB, and ROS	Rapid and readily observable for primary drug screening; LPS may exaggerate inflammation, and the model remains limited to early atherosclerosis-like changes	[51,80]
Inherited metabolic disease	Embryonic or larval	Transient knockdown: target-gene morpholino with mismatch-MO and/or mRNA rescue	Rapid detection of developmental, locomotor, or metabolic phenotypes; knockdown efficiency, survival, behavior, and target metabolites are assessed	Suitable for early functional screening; off-target and transient effects may differ from stable mutants, so independent validation is essential	[53,81]
	Larval or adult	Gene knockout: stable CRISPR/Cas9, TALEN, or ZFN models, such as *dldh* deficiency or propionyl-CoA metabolic defects	Substrate/toxic-metabolite accumulation, abnormal mitochondrial structure and function, impaired locomotion, and multiorgan metabolic abnormalities; validated by LC-MS/MS, enzyme activity, and rescue	Suitable for pathogenesis and therapeutic validation; requires consideration of paralogs, genetic compensation, developmental lethality, and incomplete coverage of the patient phenotype	[3,7,53,71,82]
	Larval or adult	Patient-variant transgenic/complementation model: transient or macrophage-specific expression of human *GBA1* N370S RNA/transgene in a *gba1*-deficient background	N370S fails to rescue macrophage lysosomal storage or resistance to mycobacterial infection; validated using lipid substrates, macrophage function, and rescue assays	Suitable for allele-specific and cell-specific effects; not a precise knock-in at the zebrafish orthologous locus and cannot be equated with the patient’s systemic genotype	[56]

Note: The table presents representative models and is not an exhaustive list of all available protocols.

**Table 2:** Minimum Validation Criteria, Minimum Reporting Requirements, and Limits of Inference for Major Zebrafish Models of Metabolic Diseases.

Model Category	Minimum Validation Criteria	Minimum Reporting Requirements	Limits of Inference When the Minimum Criteria Are Not Met	References
Obesity and dyslipidemia	(1) Abnormal body weight, BMI, or body-shape measures; (2) increased visceral adiposity, whole-body lipid, or ectopic lipid deposition in liver, muscle, or other sites; (3) abnormal lipid-metabolism measures such as TG or TC.	Strain/genetic background; developmental stage or age; sex (when determinable). Dietary models: diet composition (including fat/cholesterol source and content), feeding amount/frequency, and intervention duration. Genetic/combined models: genotype or transgenic background and relevant additional intervention conditions.	Weight gain alone should be described only as a growth or body-weight phenotype and does not justify an obesity model. Isolated cholesterol elevation should be designated hypercholesterolemia or dyslipidemia according to the evidence.	[17,21]
Diabetes and complications	(1) Persistent hyperglycemia; (2) abnormal glucose clearance, tissue glucose uptake, insulin signaling, or pancreatic β-cell function; (3) structural or functional injury in the relevant organ when a complication is investigated.	Strain/genetic background; developmental stage or age; sex (when determinable). High-glucose/chemical models: inducing agent, concentration/dose, administration route, and exposure duration. Dietary models: feed composition, feeding amount or intake, intervention duration, and fasting status. Genetic/ablation models: genotype or transgenic line and ablation conditions.	Short-term high-glucose exposure or a single elevated glucose measurement supports only a high-glucose-exposure or hyperglycemic-injury model. Hyperglycemia without organ injury does not justify designation as diabetic nephropathy, retinopathy, or neuropathy.	[33,58,129,130,131,132]
NAFLD	(1) Hepatic lipid deposition or increased lipid droplets; (2) abnormal hepatic TG or related lipid-metabolism measures; (3) hepatic steatosis confirmed by histology or standardized lipid imaging.	Strain/genetic background; developmental stage or age; sex (when determinable). Dietary models: diet composition (including fat source/content), feeding regimen, and intervention duration. Genetic models: target genotype/allele and genetic background.	Elevated whole-body lipid alone does not establish NAFLD. If only hepatic lipid staining is altered, the conclusion should be limited to increased hepatic lipid deposition or hepatic steatosis.	[9,10,42]
NASH	(1) Fulfillment of validation criteria for hepatic steatosis; (2) evidence of hepatic inflammation; (3) evidence of hepatocellular injury.	Strain/genetic background; developmental stage or age; sex (when determinable). Dietary models: diet composition, feeding regimen, and intervention duration. Chemical models: compound, concentration/dose, and exposure duration. Combined models: conditions and sequence of each intervention.	Hepatic lipid deposition without inflammation and hepatocellular injury should be designated NAFLD or hepatic steatosis rather than NASH. Without collagen or extracellular-matrix deposition, the model cannot additionally be designated hepatic fibrosis.	[42,135,136]
Atherosclerosis	(1) Vascular lipid deposition; (2) evidence of endothelial injury, endothelial dysfunction, or lipoprotein oxidation; (3) vascular inflammation such as macrophage or neutrophil recruitment.	Strain/genetic background; developmental stage or age; sex (when determinable). Dietary models: diet composition, cholesterol/fat content, feeding regimen, and intervention duration. Genetic models: genotype and genetic background. Combined models: additional inflammatory stimulus, dose, and exposure duration.	Short-term larval models generally support only early atherosclerosis-like changes and cannot be extrapolated to mature plaques, plaque rupture, thrombosis, or clinical cardiovascular and cerebrovascular events.	[2,80,106]
Inherited metabolic disease	(1) A defined genotype or loss of target-gene function; (2) a disease-specific metabolite, enzyme-activity defect, or key pathway abnormality; (3) organ, functional, or behavioral phenotypes relevant to the human disease.	Strain/genetic background; developmental stage or age; sex (when determinable). Genetic models: target gene/variant, allele, zygosity, and genetic manipulation strategy. Complementation/transgenic models: introduced construct, expression context, and rescue/comparison design.	Developmental, locomotor, or morphological abnormalities alone do not establish a specific inherited metabolic disease. Expression of a human variant without functional comparison also cannot be equated directly with the patient’s systemic pathogenic mechanism.	[3,53,82,140]

Note: The criteria in this table constitute a proposed framework based on currently available evidence and do not represent a unified international consensus standard. The minimum reporting requirements were informed by zebrafish study-design guidelines and evidence that sex, diet composition, and feeding conditions can influence metabolic phenotypes and experimental reproducibility [19,71,72,73].

## Data Availability

No new data were created or analyzed in this study. Data sharing is not applicable to this article.

## Supplementary Materials

The following supporting information can be downloaded at: https://www.mdpi.com/article/10.3390/biomedicines14081857/s1, Table S1: Representative therapeutic studies in zebrafish models of metabolic diseases.

## Abbreviations

AMPK	AMP-activated protein kinase
ApoB-LPs	Apolipoprotein B-containing lipoproteins
ATP	Adenosine triphosphate
BMI	Body mass index
CRISPR/Cas9	Clustered regularly interspaced short palindromic repeats/CRISPR-associated protein 9
HCD	High-cholesterol diet
HFD	High-fat diet
IL-1β	Interleukin-1 beta
IL-6	Interleukin-6
LPS	Lipopolysaccharide
MCP-1	Monocyte chemoattractant protein-1
mTOR	Mechanistic target of rapamycin
NAFLD	Nonalcoholic fatty liver disease
NASH	Nonalcoholic steatohepatitis
NF-κB	Nuclear factor kappa B
PPARα	Peroxisome proliferator-activated receptor alpha
ROS	Reactive oxygen species
STZ	Streptozotocin
TNF-α	Tumor necrosis factor alpha
UCP1	Uncoupling protein 1
