# Supplementary material for: A Framework for Selecting Zebrafish Models of Metabolic Diseases: Modeling Strategies, Phenotypic Validation, Mechanistic Investigation, and Efficacy Evaluation
Source: Biomedicines. 2026 Aug 18;14(8):1857. doi: 10.3390/biomedicines14081857 (PMC13509993; doi:10.3390/biomedicines14081857)
Supplement: Supplementary file 1 [file biomedicines-14-01857-s001.zip › biomedicines-4497316-supplementary.pdf]

Table S1. Representative therapeutic studies in zebrafish models of metabolic diseases

Disease category	Zebrafish model	Stage	Representative intervention	Major efficacy endpoints	Main findings	Support from other models	References
Obesity and dyslipidemia	Diet-induced overfeeding model	Adult	Globin digest	Plasma TG and TC, visceral-fat volume, and UCP1-associated transcriptomic signatures	Significantly reduced plasma TG and TC; visceral-fat volume tended to decrease, with evidence of UCP1-associated pathway activation	Validated in HFD-fed mice and 3T3-L1 adipocytes	[141]
	Short-term HFD-induced obesity model	Adult	Olivetol	Body weight, TG, TC, HMG-CoA reductase activity, liver enzymes, and liver histology	Dose-dependently reduced body weight, TG, and TC and improved liver enzymes and hepatic lipid deposition	Effects on weight, glucose/lipid metabolism, and islet inflammation validated in HFD-fed C57BL/6, db/db, NOD, and *Ldlr*−/− mice	[142,143]
	HFD plus sleep-deprivation obesity/NAFLD model	Larval	Melatonin	Body weight, hepatic lipids, cholesterol, inflammatory mediators, bile acids, and NF-κB signaling	Reduced lipid accumulation and metabolic inflammation and restored metabolic homeostasis	Attenuation of hepatic steatosis validated in HFD-fed mice and HepG2 cells	[28,144]
Diabetes and complications	Chronic diabetic-wound model induced by oral STZ plus alloxan	Adult	Metformin	Blood glucose, wound closure, inflammatory signaling, cell-cycle pathways, and proliferation	Partly restored proliferative and metabolic pathways and reduced persistent wound inflammation	Topical metformin accelerated healing in an STZ-induced diabetic-rat wound model	[36,145]
	HFD-induced type 2 diabetes model	Adult	Tirzepatide	Body weight, BMI, blood glucose, learning and memory, oxidative stress, and inflammatory endpoints	Improved glucose metabolism and cognitive behavior and reduced oxidative stress and neuroinflammation	Improved cognitive impairment through the SIRT3-NLRP3 axis in HFD-fed mice	[146,147]
	Transgenic conditional β-cell ablation and regeneration model	Larval	Adjudin	Insulin expression, β-cell calcium responses, blood glucose, and hepatic glucose uptake	Promoted β-cell functional maturation and hepatic glucose uptake and improved glucose homeostasis	Validated in mouse islets, db/db mice, and human hepatocyte spheroids	[129]

Disease category	Zebrafish model	Stage	Representative intervention	Major efficacy endpoints	Main findings	Support from other models	References
	Diabetes model induced by alloxan plus sucrose	Adult	Corn-silk extract	Blood glucose, transcriptomic differentially expressed genes, and sterol-biosynthesis genes: *fdft1*, *lss*, and *cyp51a1*	Lowered blood glucose and downregulated sterol-biosynthesis genes	Validated in Goto-Kakizaki diabetic rats	[148]
NAFLD	HFD-induced hepatic-steatosis model	Larval	Chlorogenic acid; isochlorogenic acid A; isochlorogenic acid B (tested separately)	Body weight, TG, TC, LDL-C, HDL-C, lipid staining, and PPARα/ACC proteins	Reduced circulating lipids and lipid deposition and modulated PPARα/ACC-associated lipid metabolism	Validated in oleic-acid-treated HepG2 cells	[149]
	HFD-induced hepatic steatosis with mitochondrial dysfunction	Larval	Procyanidin B-type trimers C1 and C2 (tested separately)	TG, TC, LDL-C, HDL-C, GSH, SOD, MDA, liver histology, and AMPK/mTOR-associated mRNAs	Reduced dyslipidemia and hepatic lipid accumulation, improved oxidative stress, and modulated lipid-metabolism genes	Validated in oleic-acid-treated HepG2 cells	[150]
	Short-term HFD-induced hepatic-steatosis model	Larval	Senkyunolide I	Hepatic lipids, liver volume, histology, PPARα, and CD36	Reduced hepatic lipid deposition and steatosis through a mechanism associated with PPARα activation	Validated in human hepatocytes and a hamster fatty-liver model	[151]
NASH	Liver-specific miR-7a-sponge transgenic model for functional inhibition of miR-7a	Larval	PPARγ antagonist; miR-7a mimic (tested separately)	Hepatic TG, lipid deposition, inflammatory mediators, endoplasmic-reticulum stress, and YY1-associated pathways	Reduced steatosis and inflammation and reversed the NASH phenotype caused by miR-7a deficiency	Not reported	[95]
	NASH-like model induced by HFD plus fructose	Larval	Bifidobacterium 1007478; indole-3-lactic acid (tested separately)	TG, TC, ROS, MDA, inflammatory mediators, histology, and AhR signaling	Reduced steatosis, oxidative stress, and inflammation through an AhR-dependent mechanism	Indole-3-lactic acid reduced hepatic lipid accumulation in HepG2 cells and HFD/HSD-fed mice	[136,152]
Atherosclerosis	HCD-induced early atherosclerosis model	Adult and larval	Fish oil	Vascular lipid deposition, circulating lipids, and expression of *hadhaa* and *hadhb*	Reduced vascular lipid deposition and improved fatty-acid β-oxidation-related endpoints	Omega-3 reduced aortic lipid area and wall thickness in atherosclerotic mice	[153,154]

Disease category	Zebrafish model	Stage	Representative intervention	Major efficacy endpoints	Main findings	Support from other models	References
	apoeb knockout plus HFD model of dyslipidemia	Larval	Simvastatin; ezetimibe; Xuezhikang (tested separately)	Whole-body and vascular lipids and macrophage/neutrophil recruitment	All three interventions reduced hyperlipidemia; Xuezhikang also inhibited inflammatory-cell recruitment	*Apoe*−/− mice and HCD-fed rats independently support the antiatherosclerotic effects of simvastatin, ezetimibe, and Xuezhikang	[2,155-157]
	HCD plus LPS atherosclerosis model	Larval	Atorvastatin; aspirin; vitamin C (tested separately)	Cholesterol deposition, inflammatory cells, vascular lipids, ROS, SOD, MDA, and plaque-like lesions	Produced differing lipid-lowering, anti-inflammatory, and antioxidant effects depending on the induction method	Mouse studies support improved plaque stability with atorvastatin and slower lesion progression with low-dose aspirin; results for vitamin-C-containing antioxidant combinations vary by strain	[51,158-160]
Inherited metabolic disease	*dldh* loss-of-function model of DLD deficiency	Larval	Low-dose thiamine; probucol (tested separately)	Locomotor performance, lactate/pyruvate, liver pathology, and mitochondrial structure	Each monotherapy improved locomotor phenotypes, but biochemical rescue was limited	Thiamine improved survival and reduced mitochondrial stress in *dld-1*-deficient Caenorhabditis elegans; probucol was not evaluated	[3,161]
	rrm2b loss-of-function model of mitochondrial-DNA depletion	Larval	Combined dAdo + dGuo supplementation with EHNA	Mitochondrial-DNA copy number, lactate, and locomotor performance	Increased mitochondrial-DNA copy number, reduced lactate, and improved locomotor phenotypes	Independent cell study: related deoxynucleotide-monophosphate supplementation increased mtDNA in patient-derived myotubes and myogenically converted fibroblasts, including *RRM2B*-mutant cells	[140,162]
	Genetic/pharmacological models of multiple respiratory-chain defects	Adult and larval	Zagociguat	Survival, locomotor performance, oxygen consumption, and respiratory-chain function	Improved locomotion and mitochondrial function in selected models, with genotype-dependent efficacy	Independent cell study: sGC-cGMP pharmacology of CY6463 (zagociguat) validated in HEK293 cells and primary rat neurons	[163,164]

Note: The table presents representative models and is not an exhaustive list of all available approaches. Gene symbols first introduced in Table S1 are defined as follows: low-density lipoprotein receptor (*Ldlr*), a protein-coding gene encoding a cell-surface receptor required for LDL uptake and clearance; farnesyl-diphosphate farnesyltransferase 1 (*fdft1*), a protein-coding gene encoding the endoplasmic-reticulum enzyme squalene synthase; lanosterol synthase (*lss*), a protein-coding gene encoding an oxidosqualene cyclase; cytochrome P450 family 51 subfamily A member 1 (*cyp51a1*), a protein-coding gene encoding sterol 14α-demethylase; hydroxyacyl-CoA dehydrogenase trifunctional multienzyme complex subunit alpha a (*hadhaa*) and hydroxyacyl-CoA dehydrogenase trifunctional multienzyme complex subunit beta (*hadhb*), protein-coding genes encoding subunits of the mitochondrial trifunctional protein involved in long-chain fatty-acid β-oxidation; apolipoprotein E (*Apoe*), a protein-coding gene encoding a lipid-transport apolipoprotein that mediates lipoprotein clearance; dihydrolipoamide dehydrogenase (*dld-1*), a *C. elegans* protein-coding gene encoding the mitochondrial E3 enzyme shared by several dehydrogenase complexes; and ribonucleotide reductase M2 b (*rrm2b*), a protein-coding gene encoding a ribonucleotide-reductase subunit required for deoxynucleotide supply and mitochondrial-DNA maintenance.
